# International health and wellness of online child sexual exploitation police personnel: individual, management, and organizational realms of responsibility

**DOI:** 10.3389/fpsyg.2023.1155733

**Published:** 2023-05-19

**Authors:** Tijana Simonovska, Roberta Sinclair, Kristin Duval

**Affiliations:** Royal Canadian Mounted Police (RCMP), Ottawa, ON, Canada

**Keywords:** health and wellness, online child sexual exploitation, police personnel, psychological distress, mitigation tools and strategies, child sexual exploitation material

## Abstract

Recognizing the need to better understand the operational and organizational stressors as well as the short- and long-term effects of working in the area of online child sexual exploitation (OCSE), the Virtual Global Taskforce international health and wellness study engaged current and former police personnel (inclusive of civilian and sworn officers) working in this area of specialization. Through the use of an online survey, this study engaged current (*n* = 516) and former (*n* = 126) personnel and focused on a thematic exploration of several topics of significance: job demands (sources that create distress), general health outcomes (including specific changes since joining or leaving an OCSE unit), health and wellness policies (beneficial aspects, suggested improvements, and desired components if no policy was in place), job resources (sources of positive energy) and personal resources and coping strategies. Recognizing that the health and wellness of police personnel working in OCSE units is not a “one-size-fits-all” approach, this study proposes a unique approach to understanding these impacts, effects and stressors by showcasing the findings across three distinct realms of responsibility: the individual level, the management level and organizational level highlighting the ways in which these work toward creating a holistic approach to safeguarding and maintaining the health and wellness of police personnel.

## Introduction

Policing has long been regarded as a profession that has its own unique set of stressors and challenges (Abdollahi, 2002; Webster, 2013; Carleton et al., 2018; Purba and Demou, 2019; Ricciardelli et al., 2020). Police personnel are often exposed to various job-specific operational stressors, for example dangers and risks inherent to police work, traumatic events, as well as organizational stressors, such as challenges related to increased workloads and staff shortages (Webster, 2013; Carleton et al., 2018; Purba and Demou, 2019; Ricciardelli et al., 2020). While it has been generally noted that police personnel may experience a range of symptoms in response to such stressors, much of the existing literature that explores operational stressors tends to focus on critical incident stress responses or the stressors that are considered as inherent to police work itself (Stinchcomb, 2004; Colwell, 2005; Purba and Demou, 2019; Ricciardelli et al., 2020). In more recent literature, there has been some exploration on the impacts of everyday stressors that police personnel may experience and the potential negative impacts it may have to their physical and mental wellbeing (Greinacher et al., 2019; Hofer and Savell, 2021; Eikenhout et al., 2022). While this exploration has shed some light on the aspects of policing that are more intrinsic to the profession, there tends to be less research devoted to understanding chronic stressors such as working in high-risk areas where there may be long term exposure to sensitive and traumatic material such as child sexual exploitation material (CSEM), despite the potential for significant effects on the health and wellness of personnel.

This gap in understanding was acknowledged among members of the Virtual Global Taskforce (VGT), an international alliance comprised of law enforcement, non-governmental and industry partners dedicated to protecting children from online child sexual exploitation and transnational child sex offenses. In an effort to address this gap, the Royal Canadian Mounted Police (RCMP), on behalf of the VGT, led an international study that sought to shed light on the unique stressors and challenges of working in online child sexual exploitation (OCSE) units and to identify ways in which these can be mitigated. The primary research question that guided this study was: what are the operational and organizational stressors and long-term and short-term effects experienced by police personnel working in OCSE units? Additionally, several sub-questions addressed the impacts of working in this area (both positive and negative) as well as mitigation strategies related to working in the area of OCSE.

### Literature overview

Operational stressors or those stressors considered to be intrinsic to policing, such as frequent exposure to and/or involvement in dangerous situations, exposure to traumatic and difficult events as well as the risk associated with the job, have been recognized for their potential to lead to various unique challenges and impacts. When coupled with organizational stressors, such as inadequate resources, heavy workload, lack of support, time pressure and strict management, the effects of operational stressors can be exacerbated and may result in risk to the health and wellbeing in police personnel (Abdollahi, 2002; Carleton et al., 2018; Purba and Demou, 2019; Ricciardelli et al., 2020).

The effects of chronic stressors as they relate to policing generally tend to be explored in the context of those aspects of the job that are considered to be intrinsic to the occupation (i.e., in the context of critical incident responses). Some of the available literature suggests that chronic stressors (which may be a combination of operational and/or occupational stressors and exposures) may result in various challenges and impacts experienced by police personnel (Powell et al., 2014; Purba and Demou, 2019). However, very limited literature has explored the effects of chronic stressors unique to the work in high-risk or specialized areas where police personnel may experience frequent and prolonged exposure to sensitive and traumatic material. While it is recognized that the degree to which impacts from these stressors are experienced and/or may affect an individual’s mental health and wellbeing may vary, generally, within the literature it has been observed that police personnel may experience various impacts ranging from exhaustion, burnout, sleep disturbances, fatigue, depression, anxiety, post-traumatic stress disorder (PTSD), secondary traumatic stress (STS) disorder, to problems with interpersonal relationships, work conflicts, difficulties with decision making and so on (Powell et al., 2014; Greinacher et al., 2019; Purba and Demou, 2019; Eikenhout et al., 2022). The extent to which these impacts and stressors stem from prolonged exposure to chronic stressors such as those resulting from working in OCSE units represents a gap within the existing literature that this study set out to address.

## Method

### Participants and recruitment

An initial review of the existing literature demonstrated that while there is research that has focused on OCSE police personnel specifically, there are existing gaps in understanding the challenges, stressors and both the positive and negative impacts of working in this area. To address these gaps, an online survey was developed which sought to collect information relevant to the realities and experiences of both current and former personnel. The survey methodology proved to be the most appropriate in conducting this large-scale study. The survey method allowed for a large number of participants to be reached while ensuring participant anonymity. The survey design and dissemination strategy allowed for individual responses, which were often of very sensitive nature, to remain confidential and not be traced back to an individual as the survey link was open and not associated to any one individual. The survey questions were largely based on the questions used in a 2014 Dutch research study conducted by Impact (the Dutch knowledge and advice center for psychosocial care concerning critical incidents), and commissioned by the Dutch National Police (Gouweloos-Trines et al., 2018). The survey was drafted in English and translated into five languages (French, Arabic, Dutch, German and Spanish).

Participants were recruited through the VGT police member agencies. Through a purposive sampling strategy, each participating agency was responsible for identifying personnel who met the inclusion criterion: personnel who at the time of the study were working or had worked in an OCSE unit, in any capacity and for any period of time. Following the established sample of eligible participants, a snowball sampling technique was employed wherein VGT police member agencies were responsible for further distribution of the survey to domestic law enforcement partners. The invitation to participate was shared via email and included a link to the online survey.

The total number of survey responses, *n* = 642, included *n* = 516 completed surveys from current personnel and *n* = 126 completed surveys from former personnel. The incomplete surveys were not accounted for as part of the total number of surveys completed, nor were the partial responses analyzed or included in the results of the study.

### Procedure

Participants were provided with a brief introduction to the project and were required to consent to participating in the study. Participants also had the opportunity to select the language of their choice and to provide their responses anonymously.

At the start of the survey, demographic information was collected about age, sex, marital status, whether they had children under 18, years of police experience, category of employment, years of experience in OCSE, primary position of employment, area of specialization, and exposure to and amount of time viewing CSEM.

Following that, participants were asked to respond to a series of closed-ended questions from a drop-down template, as well as via open-ended questions that allowed participants to offer their perspective and experience as it related to their work and the following themes of interest: job demands (sources that create distress), general health outcomes (including specific changes since joining or leaving an OCSE unit), health and wellness policies (beneficial aspects, suggested improvements, and desired components if no policy was in place), job resources (sources of positive energy) and personal resources and coping strategies.

Given the sensitivity of this study there were several ethical considerations implemented. Firstly, due to the personal nature of the questions asked as part of this survey, participant consent was required. Procedurally, participants were required to provide consent at the onset of the survey in order to be able to compete it. Once consent was provided, the participants were presented with the survey questions and able to complete them anonymously. Anonymity and confidentiality were further assured by the design of the survey link which was not traceable back to any individual. The completed surveys were compiled by the RCMP Survey Center. Surveys completed in a language other than English were translated by elected members of the home agencies of the responding participants. The data analysis was competed in English and included translated copies of the survey responses submitted in a language other than English. All original transcripts were retained, including those completed in languages other than English, for quality assurance and to ensure that conceptual equivalence was achieved throughout the data analysis process. The responses were compiled into two master Excel spreadsheets, one for the responses completed by current personnel and the other for the responses completed by former personnel and were made available to the researchers at the Program Research and Development Unit of the Strategic and Operational Services section, of the Sensitive and Specialized Investigative Services branch, of the Royal Canadian Mounted Police for analysis. The data itself was password protected and stored electronically in designated folders to which only the researchers working on this study had access to. Hard copies of the data that were utilized for some of the qualitative analysis were also stored in a secure location to which only the researchers working on the study had access.

Lastly, given the sensitivity and personal nature of the questions that were asked as part of the survey, each participating country was asked to provide agency-specific contact information and/or resources for their respective employee assistance programs which was included within the online survey and made available to the participants and researchers. It is also important to note that participation in the research study was voluntary and participants were able to withdraw at any point in time. There were also no benefits nor penalties associated with participation in this research study for the individual or the agency and/or organization represented.

## Analysis

Through the use of the Statistical Package for the Social Sciences (SPSS) and Microsoft Excel, questions that contained the population demographics as well as the top responses under each category were assessed and recorded. Correspondingly, through the use of qualitative content analysis, the open-ended questions were assessed. The qualitative findings were used to supplement the findings of the quantitative analysis as well as to explore variability amongst the received responses.

The implementation of a qualitative methodology allowed for an exploration of various themes within a large qualitative data set (Berg, 2009; Nowell et al., 2017). Qualitative content analysis as a methodology proved useful for the analysis of large amount of data and allowed for more efficient organization of the data into themes and categories that simplified the analysis process. The qualitative analysis was completed using a mix of both deductive and inductive approaches. First, the main themes were generated deductively through the use of the existing section-specific themes that guided the survey. From there, through an inductive approach, the categories of analysis were established to allow for further stratification of responses. Finally, individual codes that emerged from the data itself, were also captured which allowed for variance among the individual responses to be recorded. This resulted in the responses being coded based on a general theme, general coding category and the corresponding codes.

## Results

The demographic findings offered a strong contextual background that provided insight into the two personnel groups that participated in this study. These findings indicated that most participants were married (72.2% of current personnel and 78.6% of former personnel), both current and former personnel were most likely to fall within the age range of 31–50 years old, were police officers/sworn peace officers (72.1% of current personnel and 77.0% of former personnel) engaged in (or had engaged) in general investigations (70.1% of current personnel and 73.4% of former personnel) and had been exposed to CSEM as part of their regularly assigned tasks (87.4% of current personnel and 94.4% of former personnel), and most commonly spending (or spent) between 0 and 50% of their shifts viewing CSEM. Furthermore, a wide range of years of experience in policing and in an OCSE unit was observed, and males were slightly more represented within the sample (64.9% of current personnel were male while 54.8% of former personnel were male). The extent to which this breakdown of variables is representative of the true population is unknown. A full breakdown of demographic variables is available in Table 1.

**Table 1:** Demographics—current and former personnel.

Demographic variables		Current	Former	Demographic variables		Current	Former
		Percentage of responses (%)^1^				Percentage of responses (%)	
Country	United States	34.4%	18.3%	Years of Experience in ICE	Less than 1 year	11.7%	12.9%
	Canada	18.5%	29.4%		1–2 Years	20.8%	21.0%
	Australia	10.1%	37.3%		3–4 Years	22.8%	34.7%
	United Kingdom	6.6%	4.0%		5–7 Years	18.5%	20.2%
	Switzerland	5.3%	1.6%		8–10 Years	10.9%	4.8%
	United Arab Emirates	3.9%	0.8%		Over 10 years	15.4%	6.5%
	Colombia	2.9%	3.2%	Primary position of employment	Line officer/agent	27.4%	33.9%
	Denmark	1.8%	3.2%		Internet Child Exploitation (ICE) Investigator/detective	23.3%	13.7%
	Germany	1.6%	0.0%				
	France	1.2%	0.0%		Forensics/digital and/or IT Forensics	14.4%	5.6%
	Other	5.1%	0.0%				
Sex	Male	64.9%	54.8%		First-line supervisor	12.8%	3.2%
	Female	35.1%	45.2%		Researcher	7.2%	3.2%
Current age	Under 25 years old	1.6%	0.0%		Senior management	4.1%	7.3%
	26–30	8.0%	5.6%		Technology support	1.7%	0.8%
	31–35	16.9%	12.7%		Administrative	0.2%	25.0%
	36–40	23.9%	22.2%		Other	8.9%	7.3%
	41–45	19.8%	23.0%	Area of Specialization	General investigation	70.1%	73.4%
	46–50	17.3%	13.5%		Support services	30.1%	16.9%
	51–55	7.2%	14.3%		Victim identification	26.6%	13.7%
	Over 55	5.3%	8.7%		Triage	22.5%	22.6%
Marital status	Married/common law	72.2%	78.6%		Undercover operations	21.4%	21.0%
	Single	11.4%	11.9%		Other	10.5%	8.9%
	Dating/in a relationship	11.2%	2.4%	Exposed to CSEM as part of tasks	Yes	87.4%	94.4%
	Divorced	5.3%	7.1%		No	12.6%	5.6%
Children under 18	Yes	55.5%	51.2%	Percentage of shift viewing CSEM	0–25%	62.7%	44.4%
	No	44.5%	48.8%		26–50%	21.0%	33.3%
Years of Police Experience	Less than 5 years	10.2%	1.6%		51–75%	11.7%	14.8%
	5–10 years	20.9%	19.4%		76–100%	4.6%	7.4%
	11–15 years	23.7%	24.2%				
	16–20 years	19.2%	22.6%				
	21–25 years	11.2%	12.9%				
	26–30 years	9.4%	8.1%				
	Over 30 years	5.5%	11.3%				
Category of Employment	Sworn peace officer/police officer	72.1%	77.0%				
	Civilian Member	9.2%	4.0%				
	Public Servant	8.8%	8.7%				
	Unsworn employee	3.3%	4.8%				
	Other	6.6%	5.6%				

^1^Percentage values were calculated based on the number of responses (“counts”) for the variable divided by the total number of responses submitted for that respective variable

From the various themes that were explored, participants were asked to respond to a series of closed-ended and open-ended questions that allowed them to offer their perspective and experience as it related to their work. Current personnel were asked to provide responses to the questions under each theme by reflecting on the preceding 6–8 weeks (prior to taking the survey), whereas former personnel were asked to answer the questions under each theme by reflecting on their time spent at the unit. A scale from 0 (“not at all”) to 4 (“to a very great extent”) was used. The findings below are presented based on the five highest mean values that were reported as part of the responses for each theme, however, it is important to note that while the results presented here include the top identified challenges and stressors, in some instances there were additional responses identified through the open-ended questions that further contextualized various challenges/stressors or generally the individual experience of working in OCSE.

### Job demands (sources that create distress)

Based on the five highest mean values, the job demands that created the most distress for current personnel were: work pressure, inadequate resources, policies and procedures, the learning curve when they joined the unit, and not having the proper tools and equipment at the work place (poor work facilities). Refer to Table 2 for a breakdown of responses by percentage value, mean scores, standard deviations, and the total number of responses for each job demand category for current personnel.

**Table 2:** Job demands of current personnel.

In the last 6–8 weeks, to what extent did you feel distressed from:	Scale (0 = Not at all; 4 = To a great extent)					N/A	MEAN	SD	# Responses
	Percentage of responses (%)^2^								
	0	1	2	3	4				
Work pressure	13.2%	21.0%	34.4%	22.1%	9.3%	0.0%	1.93	1.15	515
Inadequate resources	21.9%	24.4%	21.1%	17.2%	15.4%	0.0%	1.80	1.37	512
Policies and procedures	26.2%	31.1%	24.9%	12.9%	4.9%	0.0%	1.39	1.15	511
Learning curve when joined the unit	29.0%	30.0%	18.9%	11.1%	5.4%	5.6%	1.30	1.19	514
Poor work facilities	35.3%	24.2%	22.0%	12.9%	5.7%	0.0%	1.29	1.23	513
Exposure to material	31.4%	34.2%	16.8%	6.1%	4.9%	6.6%	1.13	1.11	512
Ongoing learning curve (if at unit for more than 6 months)	35.3%	25.3%	18.8%	7.5%	3.7%	9.4%	1.11	1.13	510
Difficulty maintaining a home and work balance	40.2%	27.3%	20.1%	9.6%	2.9%	0.0%	1.08	1.12	513
Unit/agency reorganization and/or restructuring	47.9%	22.0%	13.2%	10.1%	6.8%	0.0%	1.06	1.28	514
Difficult relationships with other departments within own agency	43.2%	30.7%	15.8%	6.4%	3.9%	0.0%	0.97	1.10	512
Difficult relationships with external partners	42.5%	33.3%	16.4%	4.9%	2.9%	0.0%	0.92	1.02	513
Difficulties with colleagues	46.8%	29.2%	15.2%	4.9%	3.9%	0.0%	0.90	1.08	513
Contact with victims	31.6%	13.1%	6.7%	2.7%	1.8%	44.1%	0.75	1.05	510
Identifying with a victim	39.5%	15.3%	9.2%	2.0%	2.0%	32.1%	0.70	1.00	511
Interacting with family of an offender	34.2%	13.1%	9.4%	2.2%	0.8%	40.3%	0.70	0.95	511
Difficult relationship with supervisor	65.6%	17.7%	8.6%	4.9%	3.3%	0.0%	0.63	1.05	514
Contact with offenders	40.8%	16.2%	7.2%	2.5%	1.0%	32.2%	0.62	0.92	512

^2^Percentage values were calculated based on the number of responses (“counts”) for the scale value divided by the total number of responses submitted for that respective job demand.

Additional job demands that created distress were identified which included inadequate equipment (IT) and tools further reinforcing poor work facilities to be among the top stressor that created distress for current personnel. As participant 641 mentioned: *“Inadequate resources cause the greatest distress. Not having properly functioning equipment necessary to do my job and not having the training necessary to understand some of the technical aspects of the job.*” Similarly, training and development in order to remain up-to-date with new trends, technology, investigative tools and techniques was highlighted as a stressor which is closely linked to the need to have the proper tools and equipment at the workplace.

Former personnel who had previously worked in OCSE units were also asked to identify the extent to which they felt distressed due to various job demands while they worked at the unit. Based on the five highest mean values, former personnel were the most distressed from: work pressure, inadequate resources, exposure to the material, policies and procedures and difficulty maintaining a home and work balance. Refer to Table 3 for a breakdown of responses from former personnel.

**Table 3:** Job demands of former personnel.

While you were at the unit, to what extent did you feel distressed from:	Scale (0 = Not at all; 4 = To a great extent)					N/A	MEAN	SD	# Responses
	Percentage of responses (%)^3^								
	0	1	2	3	4				
Work pressure	8.7%	20.6%	38.1%	21.4%	11.1%	0.0%	2.06	1.10	126
Inadequate resources	12.7%	27.8%	22.2%	22.2%	15.1%	0.0%	1.99	1.27	126
Exposure to material	9.5%	25.4%	35.7%	16.7%	10.3%	2.4%	1.93	1.12	126
Policies and procedures	16.1%	35.5%	29.8%	14.5%	4.0%	0.0%	1.55	1.05	124
Difficulty maintaining a home and work balance	32.8%	26.4%	18.4%	12.0%	10.4%	0.0%	1.41	1.33	125
Poor work facilities	25.4%	34.9%	24.6%	10.3%	4.8%	0.0%	1.34	1.11	126
Unit/agency reorganization and/or restructuring	36.0%	24.8%	18.4%	16.0%	4.8%	0.0%	1.29	1.24	125
Contact with victims	14.3%	27.0%	13.5%	6.3%	1.6%	37.3%	1.27	1.01	126
Learning curve when joined the unit	28.8%	32.0%	20.8%	8.0%	3.2%	7.2%	1.19	1.08	125
Identifying with a victim	26.2%	21.4%	16.7%	8.7%	1.6%	25.4%	1.17	1.10	126
Difficulties with colleagues	41.9%	25.0%	16.9%	9.7%	6.5%	0.0%	1.14	1.25	124
Difficult relationships with external partners	29.0%	39.5%	22.6%	6.5%	2.4%	0.0%	1.14	0.99	124
Difficult relationships with other departments within own agency	34.7%	32.3%	22.6%	7.3%	3.2%	0.0%	1.12	1.07	124
Interacting with family of an offender	24.2%	23.4%	14.5%	4.8%	2.4%	30.6%	1.10	1.07	124
Difficult relationship with supervisor	46.4%	17.6%	23.2%	5.6%	7.2%	0.0%	1.10	1.25	125
Contact with offenders	33.3%	18.3%	15.1%	1.6%	2.4%	29.4%	0.89	1.04	126
Ongoing learning curve (after at unit for more than 6 months)	40.0%	31.2%	14.4%	2.4%	2.4%	9.6%	0.85	0.97	125

^3^Percentage values were calculated based on the number of responses (“counts”) for the scale value divided by the total number of responses submitted for that respective job demand.

The top two most distressing job demands, work pressure and inadequate resources, were the same as those identified by current personnel. However, among the top five job demands, there was one notable difference between the two groups, the exposure to material. This observation perhaps suggests that the effects of exposure may only become truly apparent once an individual no longer works in the area, while reflecting on the stressors. Supporting findings were provided by a number of former personnel. As participant 67 noted: “*…there were certainly times where I had low motivation and questioned the work but I just want to point out that I’ve had those feelings in all the teams I’ve worked in as a police officer to a similar degree. The main impact I felt in this team was I felt more uncomfortable around children due to the exposure to the images.”* Similarly, as mentioned by participant 38: *“Yes, the exposure to the CSAM material bothered me more that I realized at the time.”* These findings are also noted in a study by Burns (2007) who highlighted that investigators reported being upset and shocked by the type of material they encountered as part of their work in OCSE, for up to a year afterwards. In the words of participant 23: “*While immersed in the work I did not feel a thing but behave unlike myself most specifically with my now ex-husband…I was in denial…I’ve been out for 5 yrs now and I suffer from anxiety, it all came to hit me at once…I lost my marriage, felt depressed and hit the biggest wall of my life…I’m better now but it took 3–4 yrs to recover*.”

### General health outcomes

Personnel who were working in OCSE units at the time of the study reported to suffer most from: a negative worldview, overly protective behaviour toward their family and children, physical ailments, insomnia or hypersomnia, and a desire to change assignments or specific tasks. Refer to Table 4 for a breakdown of responses for current personnel. Generally, most of the current personnel working in OCSE units felt that they did not suffer from negative outcomes to any great extent. In fact, these findings are consistent with what has been previously reported in several studies, that generally personnel are coping working in OCSE relatively well (Wolak and Mitchell, 2009; Perez et al., 2010; Powell et al., 2014).

**Table 4:** Negative outcomes of work of current personnel.

In the last 6–8 weeks, to what extent did you suffer from:	Scale (0 = Not at all; 4 = To a great extent)					MEAN	SD	# Responses
	Percentage of responses (%)^4^							
	0	1	2	3	4			
Negative world view	26.6%	29.4%	25.0%	13.7%	5.3%	1.42	1.17	511
Overly protective behaviour towards family and children	39.2%	22.8%	20.9%	11.5%	5.7%	1.22	1.24	513
Physical ailments	40.1%	29.4%	19.5%	8.6%	2.5%	1.04	1.08	514
Insomnia or increased sleeping patterns	55.8%	22.4%	11.9%	7.4%	2.5%	0.78	1.08	514
A desire to change assignments or specific task	62.1%	18.8%	11.3%	5.4%	2.3%	0.67	1.03	515
Personal inability to take desired leave	61.0%	19.0%	13.8%	4.9%	1.4%	0.67	0.98	515
Changes in sexual activity	63.9%	17.9%	12.8%	3.5%	1.9%	0.62	0.97	515
Psychological ailments	64.1%	20.1%	10.0%	4.1%	1.8%	0.59	0.95	512
Cognitive impairments	73.2%	15.9%	7.6%	2.7%	0.6%	0.42	0.79	511
Increased use of alcohol, drugs or medications	75.7%	16.3%	5.3%	1.4%	1.4%	0.36	0.76	514
Diminished comfort level around children	77.7%	16.6%	3.9%	1.6%	0.2%	0.30	0.64	512
Involuntary feelings of desire or arousal	88.7%	9.7%	0.8%	0.6%	0.2%	0.14	0.44	515

^4^Percentage values were calculated based on the number of responses (“counts”) for the scale value divided by the total number of responses submitted for that respective outcome.

Furthermore, some participants specified the conditions and/or symptoms that they experienced primarily included physical or emotional symptoms such as anger, frustration, irritability, feeling overwhelmed, fatigued and experiencing loss of motivation.

#### Changes since Joining OCSE Unit (Current Personnel)

Current personnel were also asked to reflect if they had noticed any changes in themselves since joining the unit through open-ended responses. For this question they were asked to reflect on the entire duration of time at the unit. Of those who provided a response to this question, 39.7% indicated that they had not noticed any changes in themselves, while 60.3% had noticed change(s) since joining the unit. From the responses, most commonly reported changes were related to effects on their personal life and included becoming cynical/skeptical, becoming more cautious or overprotective of their children (or other people’s children), and having a negative worldview. As noted by participant 257: “[I am] *more protective for my own family/kids, more involved in preventive informational work for kids/youth, a bit more negative “world view” for human (evil) capabilities in general terms, increased appreciation of good; good deeds, good people* etc.*”* These changes could be further explained as the result of vicarious traumatization, which involves “internal changes in core beliefs, identity, needs and wants, relationships, and view of others” (Krause, 2009: 24) and has been noted as one of the potential impacts of working in OCSE units (Burns, 2007; Wolak and Mitchell, 2009; Bulmer, 2010; Whelpton, 2012). As participant 512 indicated: “*I feel uncomfortable when I see older men holding a child. When a child screams at the grocery store, I hear abuse. When I think of natural disasters, I think of children being sexually exploited for survival. When I see poor families, I think of the abuse this child may be experiencing. I am much more aware of the prevalence of this crime and as a result, see this crime everywhere I go*.” However, while some participants listed some of the challenging changes they had experienced, others noted positive changes in themselves since joining the OCSE unit. Most commonly, these changes included increased motivation, greater confidence in their work, being more empathetic and open minded, and feeling proud of the work they had done. As participant 204 indicated: “*I am happier in this role and I feel that I am contributing to our communities by doing this type of work, it is very rewarding.”* These positive findings are consistent with the literature which reports that despite the challenging nature of OCSE work, investigators working in this area have a sense of accomplishment and find great satisfaction in their work (Burns et al., 2008; Wolak and Mitchell, 2009; Bulmer, 2010; Perez et al., 2010; Whelpton, 2012; Bourke and Craun, 2013).

Similarly, former personnel reported suffering most from: a negative worldview, overly protective behaviour toward their family and children, changes in sexual activity, insomnia or hypersomnia and psychological ailments. Please refer to Table 5 for a full breakdown of responses for negative outcomes of work, for former personnel. Relating to specific changes once leaving the unit, participant 83 noted: *“I have found the support I have received since leaving to be non-existent with no follow up on any potential ongoing issues. Speaking with other people who have left the area, I have noticed that some people experienced some flashbacks* etc. *in time of stress sometimes years after leaving the area.”* Of importance is that both groups identified the same top two negative outcomes which may suggest that these particular changes often remain with individuals for some time after they leave the unit.

**Table 5:** Negative outcomes of work of former personnel.

While you were at the unit, to what extent did you suffer from:	Scale (0 = Not at all; 4 = To a great extent)					MEAN	SD	# Responses
	Percentage of responses (%)^5^							
	0	1	2	3	4			
Negative world view	15.1%	17.5%	29.4%	21.4%	16.7%	2.07	1.29	126
Overly protective behaviour towards family and children	18.3%	19.0%	23.0%	19.0%	20.6%	2.05	1.40	126
Changes in sexual activity	52.4%	15.9%	15.1%	9.5%	7.1%	1.03	1.31	126
Insomnia or increased sleeping patterns	48.4%	22.2%	13.5%	11.1%	4.8%	1.02	1.23	126
Psychological ailments	51.6%	21.4%	11.9%	10.3%	4.8%	0.95	1.22	126
Physical ailments	44.4%	23.8%	25.4%	6.3%	0.0%	0.94	0.98	126
Diminished comfort level around children	56.3%	19.0%	11.1%	9.5%	4.0%	0.86	1.18	126
A desire to change assignments or specific task	58.4%	17.6%	15.2%	2.4%	6.4%	0.81	1.18	125
Personal inability to take desired leave	61.6%	14.4%	9.6%	11.2%	3.2%	0.80	1.19	125
Cognitive impairments	67.2%	13.6%	10.4%	4.0%	4.8%	0.66	1.12	125
Increased use of alcohol, drugs or medications	73.0%	11.1%	8.7%	4.8%	2.4%	0.52	1.00	126
Involuntary feelings of desire or arousal	83.3%	11.9%	2.4%	1.6%	0.8%	0.25	0.65	126

^5^Percentage values were calculated based on the number of responses (“counts”) for the scale value divided by the total number of responses submitted for that respective outcome.

#### Changes experienced while at OCSE unit (former personnel)

Former personnel were offered the opportunity to elaborate on other outcomes and conditions that they experienced while at the unit through open-ended responses. Survey respondents most commonly reaffirmed their negative worldview, overprotectiveness and how their decisions (involving their children) may have been influenced by their work. Additionally, some respondents also acknowledged having flashbacks or associating a child or adult (seen out in public) with a victim or offender from previously viewed material. As participant 134 noted: “*I do view some men (shopping or in the public) as potential threats to children when I see a certain type of man (if he has characteristics of offenders I’ve seen in abuse material) and especially those types of men who are with children, my thoughts go to what I’ve seen and wonder if the child is a victim. I would never act on those thoughts but if there was something of concern I would intervene.*”

Former personnel were also asked if they noticed any changes in themselves since leaving the unit. Of those who responded to this question, 34.3% indicated they had not noticed any changes in themselves, while 65.7% had noticed change(s) since leaving the unit. Of those who indicated a change, some reported flashbacks, intrusive thoughts, increased cynicism and paranoia, while others highlighted that they had become less cynical and not as overprotective after leaving the unit.^1^

### Health and wellness policies

As this was exploratory research, the VGT hoped to gain some insight into how many units had established health and wellness policies, and into personnel perspective on their value. This section focused on whether or not OCSE units had a health and wellness policy in place, beneficial aspects of existing health and wellness policies and suggested improvements for said health and wellness policies (and units overall). From the responses provided by current personnel, 62.8% indicated that they did have a policy in place, while 37.2% did not.

#### Beneficial aspects (current personnel)

For those who responded that they did have a policy, they were asked a subsequent question to highlight components that they found most helpful and beneficial. The component which was reported to be most beneficial was having mandatory psychological assessments. Findings indicated that depending on the agency/unit, the frequency of the mandatory assessments varied from every 3, 6, and 12 months. As mentioned by participant 186: *“we have mandatory psychological assessment annually and such services are available to anyone, anytime. We rely heavily on peer support, and that is working well.”*

Another component which was highlighted for its helpful nature were team debriefs as a health and wellness practice within their unit. The responses that highlighted team debriefs varied and included regularly scheduled debriefs, debriefs following a major case or operation, debriefs following a stressful incident, informal, formal or, as required.

A number of participants also noted the availability of an Employee Assistance Program within their respective agency as being helpful. Peer programs as well as support from colleagues in general were also recognized by many respondents as a beneficial health and wellness resources. Lastly, pre-employment screening was also identified by a number of participants as a beneficial component of health and wellness.

#### Suggested improvements (current personnel)

Participants who had indicated that their unit/agency had a health and wellness policy were also asked to provide suggestions for improvements. Some participants indicated that mandatory psychological assessments be implemented, while those who already had assessments in place, proposed that more frequent assessments would be preferred. As noted by participant 314: “*I think psychological assessments should be more frequent and if you are advised to not be in the team for much longer then your team should endeavor to move you as soon as possible to an area of your choice.”* However, while a number of participants had noted the benefits of having a mandatory psychological assessment, some also felt that assessments should be voluntary, or upon request, rather than mandatory. Some reported skepticism of the quality of assessments and the intention behind them, where it was perceived that the assessment had little value, or was just an organizational requirement. As noted by participant 647: *“I feel that although mandatory psych assessments are in place, they may not be an accurate reflection of the employee’s well being… I feel that all employees should have their own psychologists in order to properly monitor their mental health.”* It was also pointed out that there is a need to improve response times for psychological services. As a proposed solution, hiring an on-site health practitioner (e.g., psychologist) was a suggestion made by a number of participants.

Team debriefing with (or without) a health practitioner was identified as an opportunity for improvement by a number of participants. Additionally, suggestions to involve managers and supervisors as part of these debriefs was pointed out as a beneficial component that may enhance the working relationship and aid in the understanding of how their personnel are coping.

Current personnel also suggested that the implementation of a formal pre-screening process (if one was not in place), more opportunities for team building and social activities to enhance morale and team cohesion, direct access to a physical activity room or having access to a “break” room or area would also be beneficial.

Lastly, some participants highlighted the importance of having an awareness of health and wellness in general and that enhancing the knowledge and awareness within the unit could be an opportunity for improvement, an opportunity to both break the stigma of talking about mental health and wellbeing and to normalize such conversations within the unit.

As mentioned, some participants (37.2%) indicated that at the time of the survey, they did not have a health and wellness policy in place. Of those who did not have a health and wellness policy in place, some provided suggestions on what could be helpful to them. Similar to what was mentioned by participants whose units had implemented health and wellness policies, some specified that enhanced screening would be especially beneficial at the recruitment phase. Additionally, regular psychological assessments while working at the unit, having access to a health practitioner upon request, support and flexibility to take time for physical activity during the day and having post-employment services offered (including a psychological assessment upon exit) were also suggested.

Former personnel reported that 62.7% did have a policy in place while they were at the unit, while 37.3% did not.

#### Beneficial aspects (former personnel)

For those who responded that they did have a policy, former personnel were asked to highlight the components, both formal (for example policies) or informal (for example guidelines) aspects that they found most helpful and beneficial. Similar to current personnel, mandatory psychological assessments were frequently identified as helpful, ensuring regular “check-ins” and reducing the stigma around these types of assessments since everyone had to follow the same process. As noted by participant 138: *“I believe that it was important to have mandatory assessments with a psychologist. Police officers are often to proud to ask for help otherwise or there’s a stigma associated with seeking assistance. So, the mandatory visits addressed this. It would have been nice to have team debriefs but wasn’t part of the program at that time.”* The benefits of pre-employment screenings were also highlighted as helpful components that aid and ensure fit and awareness of what the job at the unit entails. Lastly, similar to current personnel, team debriefs, team building events, social activities and peer support were recognized as important and helpful for wellbeing.

#### Suggested improvements (former personnel)

Some former personnel presented some suggestions for improvement to their health and wellness policies. These were all very similar to those identified by current personnel, such as better oversight of the mandatory assessment process as some had experienced or observed significant delays. Additionally, in terms of pre-employment screening, a few former personnel suggested that a more realistic job preview would have been helpful and could have included some information on potential impacts of working within the area of OCSE.

Improvements were also suggested for post-employment, where some noted that an exit interview or screening would have been helpful, with enhanced and continued support for a period of time after leaving an OCSE unit. As participant 122 stated: “*Upon leaving the unit the same screening should be completed, as well as asking the employee if he desires further health and wellness assistance, and will not be looked down upon by the law enforcement agency or be penalized for this*.” Extended support upon leaving would ensure that these employees have resources and safeguards in place during their transition to another role or into retirement.

Some had expressed that having opportunities for assignments or secondments to other areas of policing while not permanently leaving the OCSE unit would be beneficial in providing a “break” from the work without needing to leave permanently before being ready to.

The 37.3% of former personnel who indicated that they did not have a health and wellness policy while they worked in the OCSE unit suggested similar components that would have been helpful as the current personnel. These included: the implementation (or enhanced) screening and assessments at the recruitment phase, regular psychological assessments both on a mandatory and/or voluntary basis while working at the unit, having access to a health practitioner upon request and having post-employment services offered including a psychological assessment upon exit.

### Job resources (sources of positive energy)

Based on the five highest mean values, current personnel received the most positive energy from: humor with colleagues, results of work, support from colleagues, team cohesion, and the challenging aspect of the work. Refer to Table 6 for a breakdown of responses for each job resource category, for current personnel. Additional sources of positive energy that were highlighted included, support from family and friends and the importance of maintaining a healthy balance between work and home life through the support of colleagues and keeping an open dialogue with those who shared and understood the nature of the work. As participant 456 noted: “*Positive energy from colleagues*—*great cooperation between us, understanding each one of us and the difficulty of the cases we investigated, very supportive team*.” Moreover, while relationships with colleagues were found to be very important sources of positive energy, current personnel also highlighted the importance and benefits of having hobbies, exercise and various form of self-care practices as a way to disengage from the work. Lastly, positive investigative outcomes were highlighted as an extension of the “results of work” resource, such as offender convictions, involvement in cases and knowing the outcome, and victim safeguarding.

**Table 6:** Job resources of current personnel.

In the last 6–8 weeks, to what extent did you get positive energy from:	Scale (0 = Not at all; 4 = To a great extent)					N/A	MEAN	SD	# Responses
	Percentage of responses (%)^6^								
	0	1	2	3	4				
Humor among colleagues	1.0%	3.7%	15.9%	46.2%	33.2%	0.0%	3.07	0.85	515
Results of work	2.7%	7.5%	30.6%	39.8%	19.4%	0.0%	2.66	0.96	510
Support of colleagues	3.7%	7.8%	28.2%	41.3%	19.0%	0.0%	2.64	1.00	511
Team cohesion	4.7%	10.7%	22.9%	39.5%	22.3%	0.0%	2.64	1.08	512
Challenging area of work	2.5%	8.8%	31.6%	40.8%	16.3%	0.0%	2.59	0.95	510
Autonomy	4.7%	9.6%	29.5%	39.5%	16.7%	0.0%	2.54	1.03	509
Opportunities for personal development	12.7%	22.4%	32.6%	23.0%	9.4%	0.0%	1.94	1.16	513
Recognition and appreciation from supervisors/organization	14.1%	24.5%	28.2%	23.5%	9.8%	0.0%	1.90	1.20	511
Contact with victims and offenders	14.6%	18.7%	18.7%	10.5%	3.3%	34.1%	1.53	1.15	513
Recognition and appreciation from victims	36.1%	17.9%	22.0%	16.5%	7.5%	0.0%	1.41	1.32	509
Health and wellness support services	38.3%	22.3%	20.5%	14.5%	4.5%	0.0%	1.25	1.23	512

^6^Percentage values were calculated based on the number of responses (“counts”) for the scale value divided by the total number of responses submitted for that respective job resource.

Former personnel were also asked to identify the extent to which they got positive energy from various job resources. Based on the five highest mean values, former personnel received most positive energy from: humor with colleagues, results of work, support of colleagues, team cohesion and the challenging aspects of the work. Refer to Table 7 for a breakdown of responses for each job resource category, for former personnel. These responses were the same as those reported by the current personnel as well. Additionally, similar to current personnel, support from family and friends, support from colleagues, positive outcomes of work and engaging in hobbies, activities or exercise were most frequently identified as sources that provided positive energy. As stated by participant 66: *“A fantastic team dynamic that was a supportive culture. There was never any pressure to view on days that you were doing it tough. It was a culture of ‘being in this together’ that I found positive and supportive.”*

**Table 7:** Job resources of former personnel.

While you were at the unit, to what extent did you get positive energy from:	Scale (0 = Not at all; 4 = To a great extent)					N/A	MEAN	SD	# Responses
	Percentage of responses (%)^7^								
	0	1	2	3	4				
Humor among colleagues	2.4%	3.2%	8.7%	37.3%	48.4%	0.0%	3.26	0.92	126
Results of work	1.6%	6.3%	19.0%	32.5%	40.5%	0.0%	3.04	1.00	126
Support of colleagues	1.6%	8.7%	15.9%	42.9%	31.0%	0.0%	2.93	0.98	126
Team cohesion	4.8%	6.4%	14.4%	47.2%	27.2%	0.0%	2.86	1.04	125
Challenging area of work	4.0%	8.0%	23.2%	43.2%	21.6%	0.0%	2.70	1.02	125
Autonomy	7.3%	15.3%	29.0%	37.1%	11.3%	0.0%	2.30	1.09	124
Opportunities for personal development	10.4%	20.8%	32.8%	24.0%	12.0%	0.0%	2.06	1.16	125
Recognition and appreciation from victims	21.0%	12.9%	31.5%	22.6%	12.1%	0.0%	1.92	1.30	124
Recognition and appreciation from supervisors/organization	15.2%	24.0%	28.0%	24.0%	8.8%	0.0%	1.87	1.20	125
Contact with victims and offenders	10.4%	22.4%	24.8%	11.2%	2.4%	28.8%	1.62	1.03	125
Health and wellness support services	27.8%	25.4%	27.0%	11.9%	7.9%	0.0%	1.47	1.24	126

^7^Percentage values were calculated based on the number of responses (“counts”) for the scale value divided by the total number of responses submitted for that respective job resource.

### Personal resources and coping strategies

Current personnel reported good sense of humor, appropriate workspace set-up and equipment, focusing on positive results of work/meaningfulness of work and engaging in hobbies and leisure activities as the top five personal resources and coping strategies. Refer to Table 8 for a breakdown of responses for personal resources and coping strategies of current personnel. Some of these responses, such as humor and positive results of work were also highlighted under the job resources category which reaffirms consistency in the responses provided. Some additional insights that were shared by current personnel included, recognition for the importance of taking breaks from their work and incorporating exercise or another form of self-care such as meditation, reading or listening to music to break up the work day and decompress. As participant 647 noted: “*I take time for myself during the work day. Instead of taking a lunch break I use that time to go to the gym. I feel that health and wellness starts with yourself, so I make a conscious effort to step away from the images, videos, files, and go to the gym. It makes me feel really good, in a job/building that can be so difficult to work in.*” Current personnel also discussed the importance of flexibility as an important resource. This included having a flexible work schedule/being able to work flexible hours, as supported by their managers was said to be helpful in reducing stress.

**Table 8:** Personal resources and coping strategies of current personnel.

To what extent do you agree with the following statements: to perform well at work, it is important for me to:	Scale (0 = Strongly disagree; 4 = Strongly agree)					MEAN	SD	# Responses
	Percentage of responses (%)^8^							
	0	1	2	3	4			
Have a good sense of humor	0.4%	0.2%	2.5%	33.9%	62.9%	3.59	0.59	510
Have the appropriate workspace set-up, equipment and technology	1.0%	1.2%	4.7%	39.6%	53.5%	3.44	0.73	508
Have a stable home environment	0.2%	0.8%	5.1%	43.7%	50.3%	3.43	0.65	513
Focus on positive results of work/meaningfulness of work	0.8%	1.4%	6.1%	47.3%	44.5%	3.33	0.72	512
Engage in hobbies/leisure activities	0.8%	2.3%	7.6%	43.2%	46.1%	3.32	0.78	514
Be flexible at work	0.4%	1.8%	6.8%	49.7%	41.3%	3.30	0.71	511
Get support from partner, family or friends	1.4%	1.8%	9.4%	48.1%	39.4%	3.22	0.79	513
Be committed to my work	0.6%	1.8%	8.9%	53.3%	35.4%	3.21	0.72	514
Set boundaries and know personal limitations	0.6%	0.8%	11.3%	54.6%	32.7%	3.18	0.70	513
Be empathetic towards colleagues	0.8%	3.1%	11.9%	57.5%	26.7%	3.06	0.76	513
Have a healthy and balanced diet	0.8%	4.9%	18.8%	46.3%	29.2%	2.98	0.86	510
Keep an emotional distance from work	0.6%	7.6%	13.5%	50.8%	27.5%	2.97	0.88	512
Have control over the way images are viewed	1.2%	4.1%	26.2%	45.2%	23.3%	2.85	0.86	511
Be empathetic towards victims and offenders	2.7%	5.1%	27.8%	47.3%	17.1%	2.71	0.90	510
Be spiritual/turn to spirituality	19.2%	17.8%	31.4%	18.0%	13.5%	1.89	1.29	510

^8^Percentage values were calculated based on the number of responses (“counts”) for the scale value divided by the total number of responses submitted for that respective personal resource.

#### Workspace/environment factors that alleviate stress

Within this theme, it was also important to explore workspace/environment factors that alleviated stress. Participants reported that the workspace conditions were amongst the most important for them to be able to perform well at work. Some specific aspects of a workspace that could help alleviate potential stress were also elaborated upon through an open-ended question. Current personnel highlighted many components regarding the office layout, such as the balance between an open space that allows for proximity to colleagues and also a private space as a way of minimizing distractions and preventing inadvertent exposure to CSEM. Additional workspace related factors that were identified for their potential in reducing stress included: proximity to windows, having natural light, ergonomic equipment, access to a space or a room for informal discussions/decompressing, and access to a gym. It is also important to note that some participants indicated that they were satisfied with their current workspace/environment, as such, no changes were suggested.

Former personnel were asked the same set of questions relating to personal resources and coping strategies and workspace/environment factors that alleviate stress. Former personnel reported that in order to have performed well at work it was most important to have a good sense of humor, to focus on the positive results/meaningfulness of the work, have a stable home environment, have the appropriate workspace set up/equipment and engage in hobbies and leisure activities, all of which were also mentioned by the current personnel group. Former personnel also recognized additional resources that helped with work performance such as the importance of taking breaks and having access to a designated break area, which were also consistent with those identified by current personnel. Please refer to Table 9 for a breakdown of responses for personal resources and coping strategies of former personnel.

**Table 9:** Personal resources and coping strategies of former personnel.

To what extent do you agree with the following statements: to have performed well at work, it was important for me to:	Scale (0 = Strongly disagree; 4 = Strongly agree)					MEAN	SD	# Responses
	Percentage of responses (%)^9^							
	0	1	2	3	4			
Have a good sense of humor	0.8%	0.0%	2.4%	27.4%	69.4%	3.65	0.61	124
Focus on positive results of work/meaningfulness of work	0.8%	0.0%	4.8%	42.7%	51.6%	3.44	0.67	124
Have a stable home environment	0.8%	0.0%	7.2%	45.6%	46.4%	3.37	0.69	125
Have the appropriate workspace set-up, equipment and technology	0.8%	1.6%	5.6%	50.0%	41.9%	3.31	0.72	124
Engage in hobbies/leisure activities	1.6%	0.8%	11.2%	44.0%	42.4%	3.25	0.81	125
Set boundaries and know personal limitations	0.8%	1.6%	9.8%	48.0%	39.8%	3.24	0.76	123
Be flexible at work	0.8%	1.6%	9.6%	54.4%	33.6%	3.18	0.73	125
Be committed to my work	0.8%	0.8%	12.9%	50.8%	34.7%	3.18	0.74	124
Be empathetic towards colleagues	0.8%	2.5%	8.2%	60.7%	27.9%	3.12	0.72	122
Keep an emotional distance from work	0.8%	4.8%	9.6%	52.0%	32.8%	3.11	0.83	125
Get support from partner, family or friends	2.4%	7.3%	12.1%	39.5%	38.7%	3.05	1.01	124
Have control over the way images are viewed	1.6%	3.2%	21.0%	42.7%	31.5%	2.99	0.90	124
Have a healthy and balanced diet	1.6%	4.9%	20.3%	42.3%	30.9%	2.96	0.93	123
Be empathetic towards victims and offenders	2.4%	9.6%	23.2%	48.8%	16.0%	2.66	0.94	125
Be spiritual/turn to spirituality	18.5%	25.0%	31.5%	14.5%	10.5%	1.73	1.22	124

^9^Percentage values were calculated based on the number of responses (“counts”) for the scale value divided by the total number of responses submitted for that respective personal resource.

#### Workspace/environment factors that alleviate stress

Relating to workspace or environment factors that alleviate stress, former personnel reported that working in proximity to others, while incorporating some privacy was especially significant, as it was also reported by current personnel. As participant 32 expressed: “*We need to get out of the “cubicle environment.” Interaction among colleagues is imperative in this area of work and the office arrangements are not conducive to that. Having said that it is also important that others are not exposed to the images/videos, so a certain level of privacy must be maintained*.”

Similar to current personnel, several former personnel also highlighted the importance of having a designated office space for the OCSE unit that would prevent unintentional exposure to CSEM, the importance of natural light, and comfortable and adequate workspace.

## Discussion

The objective of this study was to better understand the operational and organizational stressors and challenges as well as the short- and long-term effects of working in the area of OCSE. While the findings confirmed that personnel working in OCSE were exposed to a wide range of challenges and stressors, it was evident that much of their experience was influenced by various operational and organizational stressors. Understanding and responding to these findings spans across three realms of responsibility: the individual, management and organizational level.

Relating to the various themes that were explored, generally the responses provided by each group aligned and often highlighted responses that were either very similar or identical (as per a comparison of the reported responses based on the five highest mean scores for the same question). This speaks to the general notions that challenges and stressors as they relate to working in OCSE tend to be experienced across a wide range of individuals and even recalled when individuals are no longer working in the unit, although there were several observations that remained to be specifically applicable to each group.

Contrastingly, while there were similarities observed in the responses, there were also a few notable differences which may be impacted by various variables such as personal circumstances, location, size of team, availability of resources, training opportunities and so on. For this exploratory study, it was beyond the scope to observe individual impacts and relational impacts across the board, nevertheless these remain of interest and should be addressed by subsequent research.

The findings of this study illustrate that working in OCSE has its unique job demands, challenges and stressors that may be experienced by individuals working in this area. Specifically, both current and former personnel reported various difficulties that are interlinked with the unique aspects of investigating online child sexual exploitation: the sensitive and difficult nature of the crime(s) investigated, the impacts of the use of technology to facilitate this crime and the complexities associated with investigating this crime type (i.e., often multi-jurisdictional in nature and constantly evolving).

Employees working in this area have relayed that some of the operational stressors such as the steep learning curve when they first join the unit, the importance of having the necessary technology and technological knowledge as well as the need for proper equipment in order to conduct these investigations have a direct impact on how well these investigations can be carried out. This may lead to much frustration and can further exacerbate any job-specific challenges or difficult outcomes, such as the potential risk associated with long-term exposure to sensitive and traumatic material. Respondents to this survey recognized the negative outcomes and effects to their health and wellbeing and relationships that may be a response or reaction to the specific stressors were exposed to while at the unit. While generally these negative outcomes were noted to be similar, there was variance among the responses which may suggest that depending on an individual’s suitability to work in this area, or specific life circumstances, the level of this risk may increase. Determining the significance of individual suitability and life circumstances are just a few of the factors that need to be further researched for their relational significance. However, these preliminary findings sufficiently highlight that recognizing the potential risk associated with working in this specific area remains something that ought to be considered and communicated at all stages of the hiring process as well as when an employee leaves the unit, and can be used to inform development and implementation of recruitment, orientation and departure procedures and policies.

Organizational stressors were also noted to impact those working in or having worked in OCSE units. Participants stated that in many instances, various policies or procedures or lack thereof, made working in this area even more challenging. The fact that these findings highlight various nuances and tensions such as the need for mandatory psychological assessments or contrastingly, advocating for voluntary psychological assessments conducted by highly trained individuals (i.e., designated on-site psychologist) offer insight into some of the challenges presented to those working at the management or organizational level of police institutions. In fact, noting that the experiences and needs of those working in these areas are so diverse, points to the need to address this issue across distinct realms of responsibility: the individual, management and organizational level.

### Recommendations for promising practices

From the observed findings, it is evident that those working in OCSE should consider implementing various strategies at the individual level to help lessen some of the stressors and challenges that result from working in this area while also endeavour to enhance the positive aspects and the overall satisfaction individuals working in this area can and do experience. These can include implementation of various CSEM viewing strategies, seeking psychological/counseling services, either as part of their mandatory psychological assessment or their own initiative, development of personal coping strategies, such as hobbies, physical exercise and seeking support from their colleagues, friends and families. As discovered through this study, and reported in available literature, many participants reported that they relied on their co-workers as their main source of support given the common work experiences they share (Coughlin, 2002; Tehrani, 2011; Powell et al., 2014; Te Brake et al., 2014; Nero et al., 2022). For additional benefit, consideration should be given to incorporating conversations relating to the importance of individually based strategies once individuals undergo pre-employment screening(s) and/or at the orientation session stage.

More specific to former personnel, extending support beyond their time within the OCSE unit was recommended, as the impacts of the work many not be truly apparent until after leaving the unit. For example, studies have shown that psychological impacts including Post Traumatic Stress Disorder (PTSD), or more specifically, in the case of OCSE personnel, Secondary Traumatic Stress (STS), a state of heightened anxiety and tension, and the preoccupation with someone else’s suffering, with symptomology similar to that of PTSD that develops as a result of indirect exposure to traumatic material or events, can unfold long after the triggering incident(s; Krause, 2009; Bourke and Craun, 2013; Te Brake et al., 2014). It can be months or even years before symptoms appear, and/or by the time their impact is recognized and addressed (Burns, 2007; Utzon-Frank et al., 2014). This was particularly evident through the greater extent of distress experienced from viewing CSEM reported by the former personnel, in comparison to what was reported by current personnel. Interestingly, current personnel did not report a great extent of distress from viewing CSEM, as it was not ranked among the top five stressors, whereas by comparison, it was ranked number three for former personnel. Highlighting this notable difference between current and former personnel, it is possible that perhaps much of the challenges experienced by current personnel were offset by the positive changes and positive aspects of their work. Current personnel noted that working in the area of OCSE contributed to increased motivation, greater confidence in their work, and feelings of pride for the work they had done. All of these reported findings remain consistent with reports in the literature that despite the challenging aspects of the work, investigators working in this area have a great sense of accomplishment and satisfaction in their work (Burns et al., 2008; Wolak and Mitchell, 2009; Bulmer, 2010; Perez et al., 2010; Whelpton, 2012; Bourke and Craun, 2013; Sinclair et al., 2015).

From the findings it was also evident that a number of the challenges and stressors resulted from various decisions at the management level. Participants noted that they were especially impacted by the mandatory psychological assessment policies enacted, enforced or lack of such policies, as well as various challenges which stemmed from resource strains, lack of appropriate workspace/environment factors, lack of proper training opportunities as well as unrealistic expectations from managers and supervisors working in OCSE.

The findings from this study demonstrate that supportive management coupled with effective health and wellness policies, including psychological assessments for the duration of a posting/unit, in addition to follow-up assessments once an employee leaves a posting/unit, stress management programs and initiatives, appropriately sized teams, optimal work environments, adequate technical equipment, as well as sufficient financial and human resources, are of utmost importance to support current but also former OCSE personnel. Available literature also points out the benefits and positive impacts police organizations have been experiencing as a result of recent changes and efforts to prioritize the mental health and wellbeing of their personnel though the development of various strategies, programs and training (Knaak et al., 2019; Hofer and Savell, 2021; Violanti, 2021). The findings of this research as well as the literature demonstrate the critical importance and need for employee wellness programs within law enforcement organizations (Knaak et al., 2019; Hofer and Savell, 2021; Violanti, 2021). These findings also highlight the critical role of managers and supervisors in both the prevention and management of the potential impacts of work-related stressors (Milner et al., 2013; Carleton et al., 2018; Farr-Wharton et al., 2021). Managers and supervisors have a key role in both mitigating stressors and impacts as they relate to operational and organizational stressors as well as in protecting and improving the well-being of their employees (Knaak et al., 2019; Farr-Wharton et al., 2021). Oftentimes these individuals represent the link between the individual and the organizational level and as such their role signifies the importance of ensuring proper communication across all three realms as well as setting the general tone on health and wellness awareness across the board. It is through the unique responsibility that managers and supervisors bear, that effective conversations around health and wellness could be used as an opportunity to break down barriers and the stigma surrounding mental health and wellbeing, resulting in the creation of positive working environments that not only normalize these conversations but also normalize individual practice of the same. In fact, the literature notes that policing leadership can challenge organizational stigma surrounding mental health and encourage employees to reach out to the appropriate resources (Cohen et al., 2019; Knaak et al., 2019). However, it must be noted that due to the responsibility placed upon managers and supervisors working in this area, organizational support and appropriate training is required to ensure that the same kind of support is extended to them as they work to support and safeguard their personnel.

At the organizational level, recognizing the role that organizations play in promoting and supporting employee wellbeing, several opportunities and responsibilities can be highlighted. Under this realm the focus remains on strategies and initiatives that organizations can adopt in order to further enhance the workplace environment. Amongst others, these include adopting health and wellbeing policies, implementing stress management programs and other employee programs, dedicated resources, supporting the implementation of mitigation tools and techniques and keeping an open dialog on the importance of mental health and wellness in the workplace (Cohen et al., 2019; Farr-Wharton et al., 2021). Recognizing that organizational stressors represented a high percentage of the causes of distress for both current and former personnel sheds insight into the larger responsibility policing organizations have towards their personnel. By focusing on ways in which organizations can enhance the emotional, physical, and psychological welfare of their personnel, organizations will not only be effective in safeguarding their personnel, mitigating various job-specific stressors but also advance personnel retention and longevity in high risk areas and encourage effective work performance. This in turn will increase the effectiveness of police personnel and the organization in serving and protecting their communities.

Finally, from these findings it was evident that working in OCSE had some unique challenges and stressors as well as many positive aspects which encourage personnel to work in this area. Given the importance of this area of policing, it remains integral that a holistic approach to health and wellness is undertaken toward the safeguarding of these employees. While there is recognition and opportunity in the distinct nature of the three identified realms of responsibility, it is noteworthy to highlight that each realm works together to create a coordinated response strategy to increasing and maintaining employee, and hence organizational wellbeing.

### Limitations

As a first of its kind, this study sought to explore the experiences of those working or those who had worked in OCSE units, from around the world. While the results derived from this study offer a wealth of information that has had true value in determining ways to manage and mitigate some of the job-specific challenges and stressors, several limitations need to be noted.

One of the limitations is related to the sample used for this study. Due to the nature of implementing snowball sampling to disseminate the survey to as many respondents as possible, there is an inability to determine the size and demographics of the total population to which the survey was disseminated to.

Another noteworthy limitation comes from the use of a survey as the methodology. Generally, surveys can only provide estimates and assumptions about the true population, limiting the extent to which the information and results obtained through the study can be generalized beyond the tested sample. Furthermore, since both personnel groups were asked to provide responses utilizing different timeframes as points of reflection (i.e., 6–8 weeks prior to completing the survey for current personnel vs. time spent at the unit for the former personnel), it is recognized that their comparability remains limited.

As a result of these approaches the generalizability of the findings may have been impacted. While these limitations are recognized, it is important to note that this study was not intended to offer conclusive evidence, but rather to offer exploratory findings in order to establish a better understanding of the positive and negative impacts of working in OCSE units while offering insight into the various stressors experienced and the ways in which these stressors can be mitigated through the development of promising practices.

### Forward look

This exploratory research provides substantial insight into the potential stressors and positive impacts that can result for police personnel working in OCSE units internationally. The findings suggest several areas in need of research, including but not limited to: the utility and design of health and wellness policies, the potential of evidence-based screening tools at the recruitment phase, the benefits of workplace design and culture in support of health and wellness and an in-depth examination of manager’s perspectives. It is critical that managers understand the impacts of this work, are trained and educated on how to supervise personnel in these areas, and are able to recognize symptoms and feel equipped to address with personnel. Additionally, the ongoing evaluation of mitigation strategies will enable an informed approach to maintaining and strengthening the health and wellness of police personnel in these areas of specialization. Future work of the VGT will focus on some of these areas to ensure that police personnel are adequately equipped with the best tools to do their important work and to take care of themselves as well.

## Conclusion

Based on the findings of this study it is evident that there are a number of operational and organizational factors that contribute to various stressors and challenges for those working in OCSE units. While the risk factors associated with this job are uniquely experienced by individuals, the findings lend insight into which protective factors can contribute to a positive work environment and can help increase employee health and wellbeing. The findings of this research can be used to inform the ways in which individuals, management and organizations can work together to strengthen the health and wellness policies and practices among VGT member agencies and beyond. Through the development of promising practices across three distinct realms of responsibility: individual-based initiatives, management-based initiatives and organizational based initiatives, the findings can be operationalized into practical approaches and strategies that work toward minimizing and mitigating the potential negative impacts of this work^2^ while also further exploring the positive impacts. It is recognized that operationalizing the findings into promising practices can be implemented based on the applicability of the same as well as the capacity and circumstances of the country, agency, unit and/or individual. Nevertheless, a united, proactive and coordinated approach to safeguarding and maintaining the health and wellbeing of those working in the area of OCSE will only enhance the collective response of better protecting and serving those who are most vulnerable—our children.

## Data availability statement

The datasets presented in this article are not readily available due to the sensitive nature of the information contained within. Questions regarding the datasets should be directed to Royal Canadian Mounted Police, Sensitive and Specialized Investigative Services, Strategic and Operational Services Section: RCMP.VGT-GIV.GRC@rcmp-grc.gc.ca.

## Ethics statement

The studies involving human participants were reviewed and approved by Royal Canadian Mounted Police Human Resources Sector Research Review Board. The patients/participants provided their written informed consent to participate in this study.

## Author contributions

TS drafted the manuscript. KD and RS collected the data. All authors participated in the analysis of the data and drafting of the original (full length) report. All authors contributed to the article and approved the submitted version.

## Conflict of interest

The authors declare that the research was conducted in the absence of any commercial or financial relationships that could be construed as a potential conflict of interest.

## Publisher’s note

All claims expressed in this article are solely those of the authors and do not necessarily represent those of their affiliated organizations, or those of the publisher, the editors and the reviewers. Any product that may be evaluated in this article, or claim that may be made by its manufacturer, is not guaranteed or endorsed by the publisher.

## Footnotes

^1^It is important to that these are self-reported findings by personnel who responded to the survey and are not based on self-report screening tool assessments.

^2^The fulsome report includes various recommendations for promising practices. The report can be requested at: RCMP.VGT-GIV.GRC@rcmp-grc.gc.ca.
